# Placental Insufficiency in Fetuses That Slow in Growth but Are Born Appropriate for Gestational Age: A Prospective Longitudinal Study

**DOI:** 10.1371/journal.pone.0142788

**Published:** 2016-01-05

**Authors:** Nadia Bardien, Clare L. Whitehead, Stephen Tong, Antony Ugoni, Susan McDonald, Susan P. Walker

**Affiliations:** 1 Department of Perinatal Medicine, Mercy Hospital for Women, Melbourne, Australia; 2 Department of Obstetrics and Gynaecology, University of Melbourne, Melbourne, Australia; 3 Translational Obstetrics Group, University of Melbourne, Melbourne, Australia; 4 School of Physiotherapy, University of Melbourne, Melbourne, Victoria, Australia; 5 La Trobe University, Mercy Hospital for Women, Melbourne, Australia; Virgen Macarena University Hospital, School of Medicine, University of Seville, SPAIN

## Abstract

**Objectives:**

To determine whether fetuses that slow in growth but are then born appropriate for gestational age (AGA, birthweight >10^th^ centile) demonstrate ultrasound and clinical evidence of placental insufficiency.

**Methods:**

Prospective longitudinal study of 48 pregnancies reaching term and a birthweight >10^th^ centile. We estimated fetal weight by ultrasound at 28 and 36 weeks, and recorded birthweight to determine the relative change in customised weight across two timepoints: 28–36 weeks and 28 weeks-birth. The relative change in weight centiles were correlated with fetoplacental Doppler findings performed at 36 weeks. We also examined whether a decline in growth trajectory in fetuses born AGA was associated with operative deliveries performed for suspected intrapartum compromise.

**Results:**

The middle cerebral artery pulsatility index (MCA-PI) showed a linear association with fetal growth trajectory. Lower MCA-PI readings (reflecting greater diversion of blood supply to the brain) were significantly associated with a decline in fetal growth, both between 28–36 weeks (p = 0.02), and 28 weeks-birth (p = 0.0002). The MCA-PI at 36 weeks was significantly higher among those with a relative weight centile fall <20%, compared to those with a moderate centile fall of 20–30% (mean MCA-PI 1.94 vs 1.61; p<0.05), or severe centile fall of >30% (mean MCA-PI 1.94 vs 1.56; p<0.01). Of 43 who labored, operative delivery for suspected intrapartum fetal compromise was required in 12 cases; 9/18 (50%) cases where growth slowed, and 3/25 (12%) where growth trajectory was maintained (p = 0.01).

**Conclusions:**

Slowing in growth across the third trimester among fetuses subsequently born AGA was associated with ultrasound and clinical features of placental insufficiency. Such fetuses may represent an under-recognised cohort at increased risk of stillbirth.

## Introduction

Fetal growth restriction is often caused by placental insufficiency and strongly associated with an elevated stillbirth risk. Of three million stillbirths occurring globally each year, over half are growth restricted. While congenital infection, chromosomal or structural abnormalities are implicated in some small for gestational age (SGA; birthweight <10^th^ centile) stillbirths, the majority of these are normally formed. Being SGA is associated with a four-fold increase in stillbirth risk compared to fetuses born appropriate for gestational age (AGA; birthweight >10^th^ centile) [1, 2]. Fetuses not recognised to be small during pregnancy are at even higher risk, with undetected fetal growth restriction associated with a further doubling of stillbirth risk [2, 3]. The population attributable risk (PAR) of fetal growth restriction to normally formed stillbirths is estimated at 22.2% overall, rising from 6.2% to 32% among those fetuses where FGR was detected, vs undetected, prior to birth [2]. Accordingly, improved detection of fetal growth restriction (FGR) has been identified as one of the top ten interventions needed to reduce the global burden of stillbirth [4].

However, it is plausible that placental insufficiency placing fetuses at increased risk of stillbirth is not restricted to those born SGA. Notably, nearly half of normally formed stillbirths are reported to be AGA. We hypothesise there may be a subgroup of AGA fetuses with placental insufficiency who display slowing of fetal growth trajectory while in utero, but do not end up with a birthweight <10^th^ centile at term, thus not classed as SGA. Such a cohort that has declined in weight centiles in late pregnancy may be an important under-recognised group with sub-optimal placental function, and at increased risk of stillbirth.

We undertook a prospective study to examine whether a decrease in growth trajectory in fetuses subsequently born AGA is associated with ultrasound and clinical features of placental insufficiency. To obtain ultrasound evidence of placental insufficiency, we measured the middle cerebral artery pulsatility index (MCA-PI) at 36 weeks. The MCA-PI is decreased when the fetus is faced with placental insufficiency and has actively responded to this relative hypoxia by redistributing blood flow to the brain [5]. This measure of adaptive fetal behaviour was considered the best available *ultrasound parameter* of placental insufficiency in near term fetuses as, (i) it has moderate predictive accuracy for acidosis in term SGA fetuses, where its use is recommended to assist with timing of delivery [6], and (ii) cerebral redistribution has been shown to correlate with development of fetal compromise in labour among both SGA and AGA term fetuses [7]. We also measured the umbilical artery Doppler waveforms in order to calculate the cerebroplacental ratio (CPR).

Fetuses that are growth restricted have less fetal reserve and are more likely to experience compromise under the hypoxic challenge of labour, prompting intervention [8]. Thus, we chose development of intrapartum fetal compromise as the *clinical parameter* most suggestive of placental insufficiency. We examined whether a slowing in growth across the third trimester among fetuses born AGA is associated with an increased risk of requiring an operative delivery for non-reassuring fetal status.

## Methods

We performed a prospective longitudinal study to assess fetal growth trajectory in women with uncomplicated singleton pregnancies where gestational age was established from a first trimester ultrasound and following a normal mid-trimester morphology assessment. Women were invited to participate in the study at 28 weeks’ gestation, on the day of presentation for a glucose tolerance test to screen for gestational diabetes. We performed an ultrasound to assess fetal growth, performed Doppler ultrasound of fetal vessels and measured the amniotic fluid index at 28 and 36 weeks’ gestation. We also collected information on delivery outcomes after they had birthed.

The study was designed to investigate whether fetuses that are AGA at term, but slow in growth trajectory show increased evidence of placental insufficiency. Hence, the *inclusion criteria* was confined to normally formed fetuses born at term (after 37 weeks gestation) with a customised birthweight greater than the 10^th^ centile. Clinicians were notified and women *excluded* from further participation in the study if the estimated fetal weight was found to be <10^th^ centile, amniotic fluid index <5^th^ centile or the umbilical artery pulsatility index was >95^th^ centile at *either* ultrasound assessment. For the remaining women, management was left to the discretion of the patient’s clinical team, who were blinded to the fetal customized centile at both timepoints. This protocol was approved by the Mercy Health Research Ethics Committee in March 2011, Ethics Approval Number R11/04. Written informed consent was obtained from all participants.

### Ultrasound assessment of fetal size and growth

Ultrasound examinations were performed by a single experienced operator at 28 weeks and 36 weeks, using a General Electric Voluson 730 (GE Medical Systems, Zipf, Austria) equipped with a 2-7-MHz linear curved-array transducer. The Estimated Fetal Weight (EFW) was derived from standard biometric planes, and a *customised weight centile* for gestational age was created using the Australian dataset of the GROW software [9] (www.gestation.net), thus creating a customised weight centile for each fetus at three time points; 28 weeks, 36 weeks and at birth. Customised standards were chosen over birthweight standards for this study, given the stronger association with adverse perinatal outcomes attributable to placental insufficiency [10, 11]. The GROW software generates a ‘term optimal weight’ based on an optimised fetal weight standard, with adjustment for non pathological factors affecting birthweight, including maternal height, weight, ethnicity, parity and fetal gender. Coefficients for the Australian dataset of GROW were informed by a local dataset; the multiple regression model has a constant to which weight is added or subtracted for each of the variables, maternal height, weight, ethnicity, parity and fetal gender.

### Ultrasound Doppler assessment

Transabdominal colour Doppler examination was used to visualize the umbilical artery (in a free loop away from abdominal or placental cord insertion sites) and middle cerebral artery (in an axial section, with the middle cerebral artery identified overlying the anterior wing of the sphenoid bone near the base of the skull). The UA-PI and MCA-PI measurements were taken during a period of fetal inactivity and apnoea with an angle of insonation as close as possible to zero. Measurements were taken in triplicate and the mean value used, and plotted against reference data [12, 13]. The CPR was calculated as the MCA-PI divided by the UA-PI.

### Birthing outcome data

Delivery outcomes and neonatal data were reviewed by a single clinician, blinded to the previous ultrasound results. Among women who underwent labour, the indication for operative delivery was reviewed by two investigators. The diagnosis of intrapartum fetal compromise was based on the obstetrician’s documentation regarding abnormal fetal heart rate patterns, abnormal intrapartum fetal blood sampling or both.

### Assessment of fetal growth trajectory

**Fig 1:**
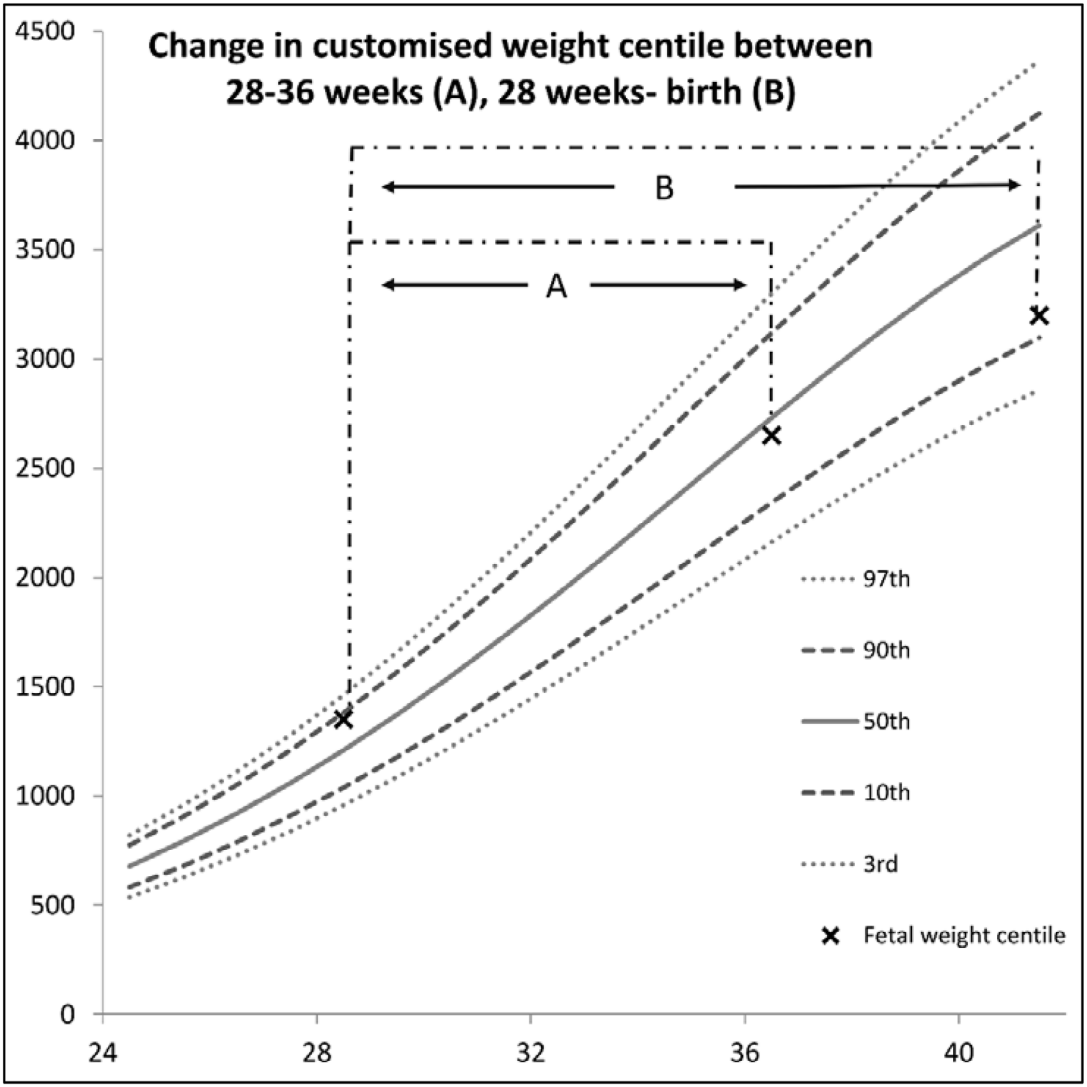
The change in customised centile between 28 weeks and 36 weeks (A) and 28 weeks and birth (B) was calculated as the later fetal weight centile minus the earlier fetal weight centile. A fetus *slowing in growth* will thus have a *negative number* to describe their weight centile change; a fetus that *maintains growth* will have a *change of zero*, and a fetus with an *increase in growth* will have a *positive number* to describe weight centile change.

Following delivery, the customised weight centile for each fetus at the three time points was used to calculate the change in weight centile across two time periods (1) between 28 and 36 weeks, and (2) between 28 weeks and birth, by subtracting the customised fetal weight centile generated at the later assessment from the one generated earlier (see Fig 1a and 1b). Hence a ‘negative’ customised centile change reflects a fall in growth across the two time points, whereas a ‘positive’ customized centile change reflects an increase in growth trajectory. The change in customised weight centile was then examined in relation to (1) Doppler findings at the final ultrasound examination prior to birth (36 weeks gestation), and (2) the need for emergency delivery (operative vaginal delivery or emergency caesarean section) for suspected intrapartum fetal compromise.

Data analysis was performed using the GraphPad Prism 5 software (Graph Pad Software, Inc., San Diego, CA, USA). To examine the relationship between slowing growth and cerebral vasodilation, the change in customised centile was correlated with the Doppler parameters performed at the final ultrasound examination. This was done firstly, with linear regression, and then by grouping the fall in customised centile between 28 weeks and birth as follows; *Group 1*: those with an increase in customised centile, no change in customised centile, or a centile fall of <20% between 28 weeks and birth (the *‘maintained growth’* group); *Group 2*: those with a centile fall of between 20 and 30% (‘*moderate decline in growth trajectory*’ group); *Group 3*: those with a centile fall of >30% *(‘severe decline in growth trajectory’* group). These thresholds were chosen recognizing that ultrasound error in generating fetal weight assessment may be as high as 10–15%. We wished to ensure the change in customised centile was sufficient to allow for ‘worst case’ error in either direction at both scans (ie. 20–30%), so minimising the chances of a false positive for ‘slowing growth’. This analysis was performed using ANOVA, with Bonferroni’s multiple comparison test. To examine the relationship between slowing growth and emergency delivery for intrapartum fetal compromise, receiver operating curves (ROC) were generated, comparing the area under the curve (AUC) of birthweight alone with change in customized centile between 28–36 weeks, and 28 weeks-birth for predicting emergency delivery for suspected fetal compromise. The predictive performance at varying growth trajectory thresholds was compared and the optimal threshold at each time point assessed using Fisher’s exact test.

## Results

52 women agreed to participate in the study and completed both ultrasound examinations. 48 had an AGA fetus at both ultrasound assessments and delivered an infant at term with customised birthweight >10^th^ centile and were included in the study. Of the 4 women we excluded, 2 delivered preterm and 2 delivered a fetus <10^th^ centile.

The mean (SD) age of the 48 participants was 30.9 (4.7) and body mass index was 23.9 (3.5) kg/m^2^. 30/48 (63%) were nulliparous. The mean (SD) customised centile was 64^th^ centile (23.6) at 28 weeks, 66^th^ centile (26.5) at 36 weeks and 53rd centile (23.7) at birth. When categorised according to centile change between 28 weeks and birth, 30/48 (63%) *maintained growth trajectory*, 8/48 (17%) had a *moderate decline in growth* and 10/48 (21%) had a *severe decline in growth*. The demographic and obstetric characteristics for these 3 groups are summarised in Table 1. The birth outcomes for this cohort were consistent with our institution’s data for the corresponding time period (caesarean section rate 32%, induction of labour rate 34% and operative vaginal delivery rate 21%).

**Table 1:** Patient demographic and delivery details according to change in growth centile.

	*Maintained Growth*	*Moderate decline*	*Severe decline*
	(<20% fall in centile between 28 weeks and birth; n = 30)	(20–30% fall in centile between 28 weeks and birth; n = 8)	(>30% fall in centile between 28 weeks and birth; n = 10)
Maternal age	29.6 (4.4)	34 (4.3)	32.4 (4.6)
BMI	23.9 (3.8)	23.9 (2.4)	23.8 (3.9)
Nulliparous	18 (60%)	4 (50%)	8 (80%)
**Ethnicity**			
Caucasian	18 (60%)	4 (50%)	5 (50%)
Asian	10 (33%)	1 (13%)	3 (30%)
Other	2 (7%)	3 (37%)	2 (20%)
**Obstetric Complications**			
Hypertensive Disorders of Pregnancy: n = 3 (6.3%)	2 (7%)	1 (13%)	0
Gestational Diabetes: n = 4 (8%)	2 (7%)	1 (13%)	1 (10%)
**Birthing Data**			
Induction of labour (IOL): n = 19 (39%)	11 (37%)	3 (37%)	5 (50%)
*Indication for IOL*: *(1) PPROM*	*1*	*1*	*3*
*(1)Hypertension in Pregnancy*	*2*	*0*	*0*
*(2)Gestational Diabetes*	*2*	*1*	*1*
*(3)Maternal Disease*	*0*	*1*	*1*
*(4)Fetal Compromise/ FGR*	*3*	*0*	*0*
*(5)Spurious labour/other*	*3*	*0*	*0*
Elective Caesarean Section (CS); n = 5 (10.5%)	5 (17%)	0	0
Emergency CS (fetal compromise or dystocia; n = 5 (10.5%)	1 (3%)	2 (25%)	2 (20%)
Operative vaginal delivery (fetal compromise/ dystocia:n = 10 (21%)	4 (13%)	3 (38%)	3 (30%)

Data presented as mean (SD), or number (%).

### Doppler parameters according to fetal growth in late pregnancy

**Fig 2:**
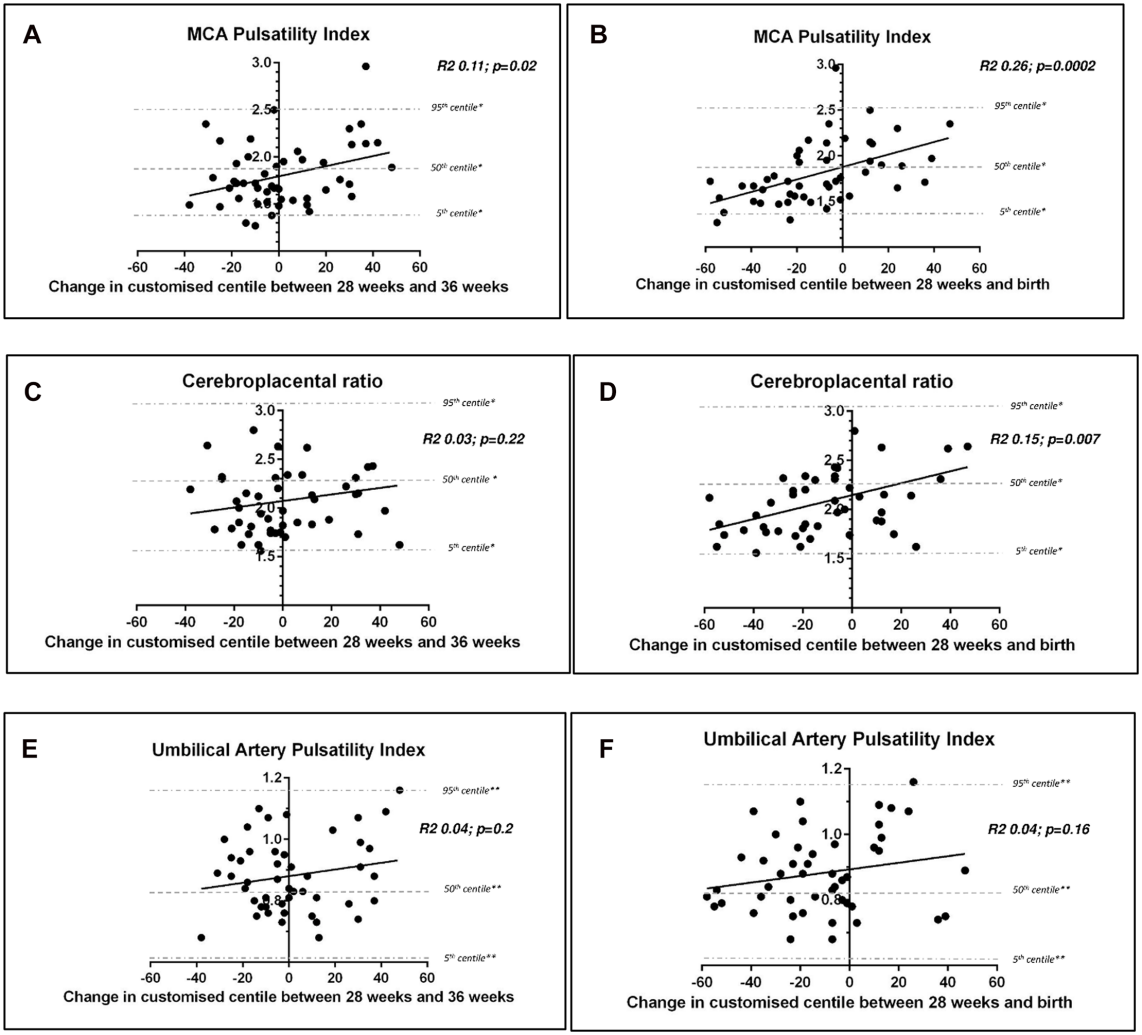
Doppler assessments performed at 36 weeks according to change in weight centile. MCA-PI according to weight centile change between the ultrasound performed at 28 weeks and 36 weeks (2a) MCA-PI according to weight centile change between the ultrasound performed at 28 weeks and birth (2b). CPR according to weight centile change between the ultrasound performed at 28 weeks and 36 weeks (2c) CPR according to weight centile change between the ultrasound performed at 28 weeks and birth (2d); UA-PI according to weight centile change between the ultrasound performed at 28 weeks and 36 weeks (2e) UA-PI according to weight centile change between the ultrasound performed at 28 weeks and birth (2f). * 5^th^, 50^th^ and 95^th^ centile for Middle Cerebral Artery Pulsatility Index and Cerebroplacental Ratio at 36 weeks gestation (12); ** 5^th^, 50^th^ and 95^th^ centile for Umbilical Artery Pulsatility Index at 36 weeks gestation (13).

We plotted the MCA-PI obtained at 36 weeks gestation with the relative change in customised weight centiles between 28 and 36 weeks (Fig 2a) and between 28 weeks and birth (Fig 2b). A significant correlation was seen between a reduced MCA-PI (suggesting centralisation of blood flow to the brain in response to placental insufficiency) and a decline in fetal growth trajectory across both time epochs: between 28 weeks-36 weeks (p = 0.02) and 28 weeks-birth (p = 0.0002). Slowing of fetal growth between 28 weeks-birth was also associated with lower CPR at 36 weeks (p = 0.007), but this association was not seen for the change in customised centile between 28–36 weeks (p = 0.22) (Fig 2c and 2d). The UA-PI was not associated with change in customised centile across either time period (Fig 2e and 2f).

**Fig 3:**
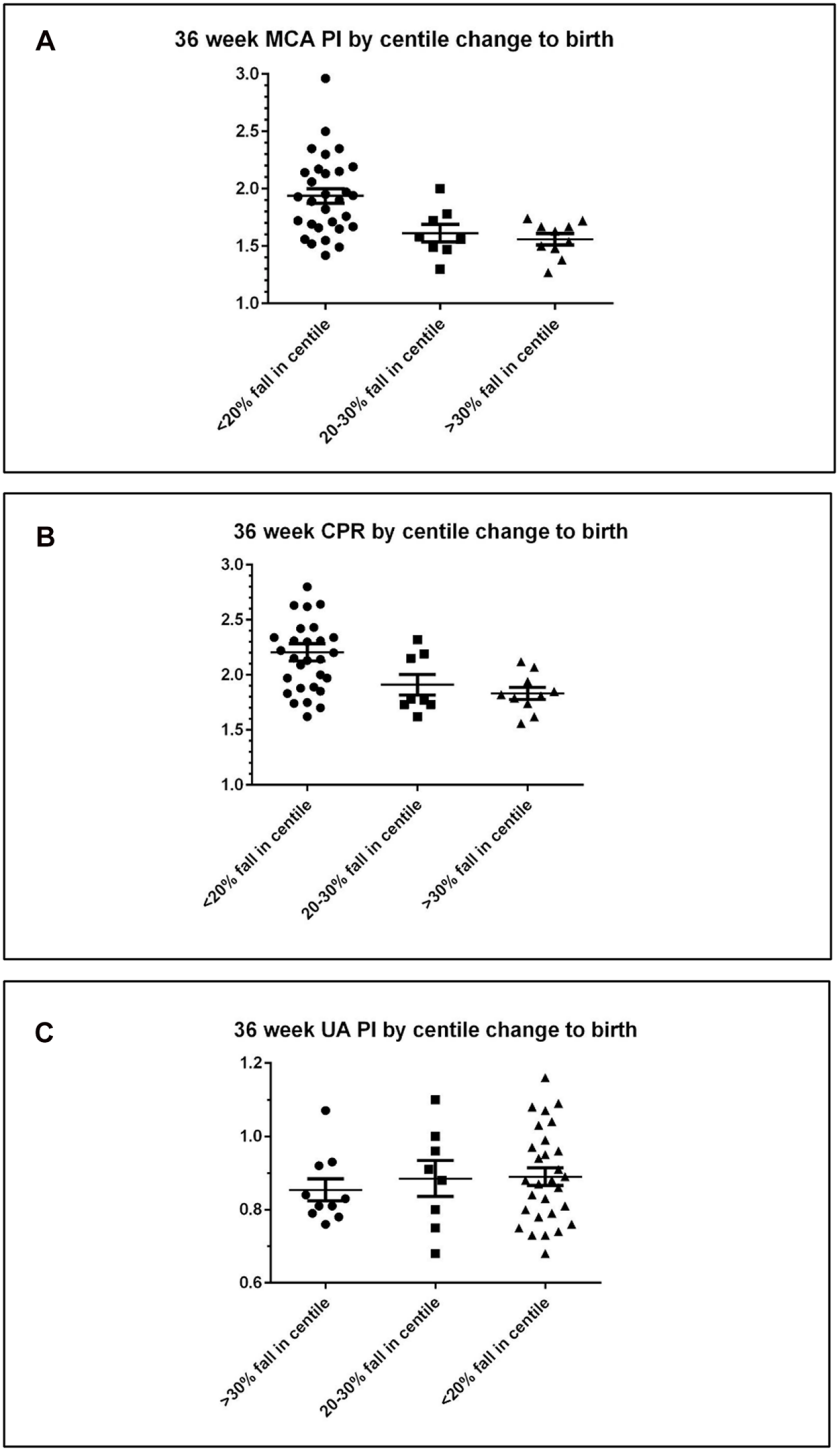
Doppler assessments performed at 36 weeks according to change in fetal growth centile by categories; (i) <20% fall in customised centile between 28 week ultrasound and birth (n = 30), (ii) 20–30% fall in customised centile between 28 week ultrasound and birth (n = 9), (iii) >30% fall in customised centile between 28 week ultrasound and birth (n = 9) for MCA-PI (Fig 3a), CPR (Fig 3b) and UA-PI (Fig 3c).

When compared to fetuses who *maintained growth* trajectory between 28 weeks and birth (n = 30), fetuses who had *moderate decline in growth* (n = 8) had a lower MCA-PI: mean (SEM) MCA-PI 1.94 (0.0.6) for maintained growth versus mean (SEM) MCA-PI 1.61 (0.08) for moderate decline in growth, p = 0.017. The MCA-PI was lower again among those with a *severe decline in growth* (n = 10): mean (SEM) MCA-PI 1.94 (0.06) for maintained growth versus mean (SEM) MCA-PI 1.56 (0.05) for severe decline in growth, p = 0.002 (Fig 3a).

This trend was also seen when examining the CPR, which was lower among those that demonstrated a *moderate decline in growth* between 28 weeks and birth compared to those that maintained growth, and was significantly lower among those that had a *severe decline in growth*; mean (SEM) CPR 2.21 (0.08) for maintained growth versus mean (SEM) 1.83 (0.06) for severe decline in growth, p = 0.02 (Fig 3b). There was no significant association with the fall in customised centile between 28 weeks-birth and the UA-PI (Fig 3c).

### Birthing outcomes according to fetal growth in late pregnancy

We next sought to obtain evidence that a slowing of fetal growth trajectory may be associated with clinical evidence of placental insufficiency. It is established that fetuses that are growth restricted due to placental insufficiency tolerate labour more poorly and have a higher incidence of operative intervention arising from non-reassuring fetal status [8]. Therefore, we examined whether AGA fetuses that have demonstrated slowing of growth across the third trimester have an increased incidence of operative delivery for suspected intrapartum fetal compromise, compared to those who maintained growth trajectory.

**Fig 4:**
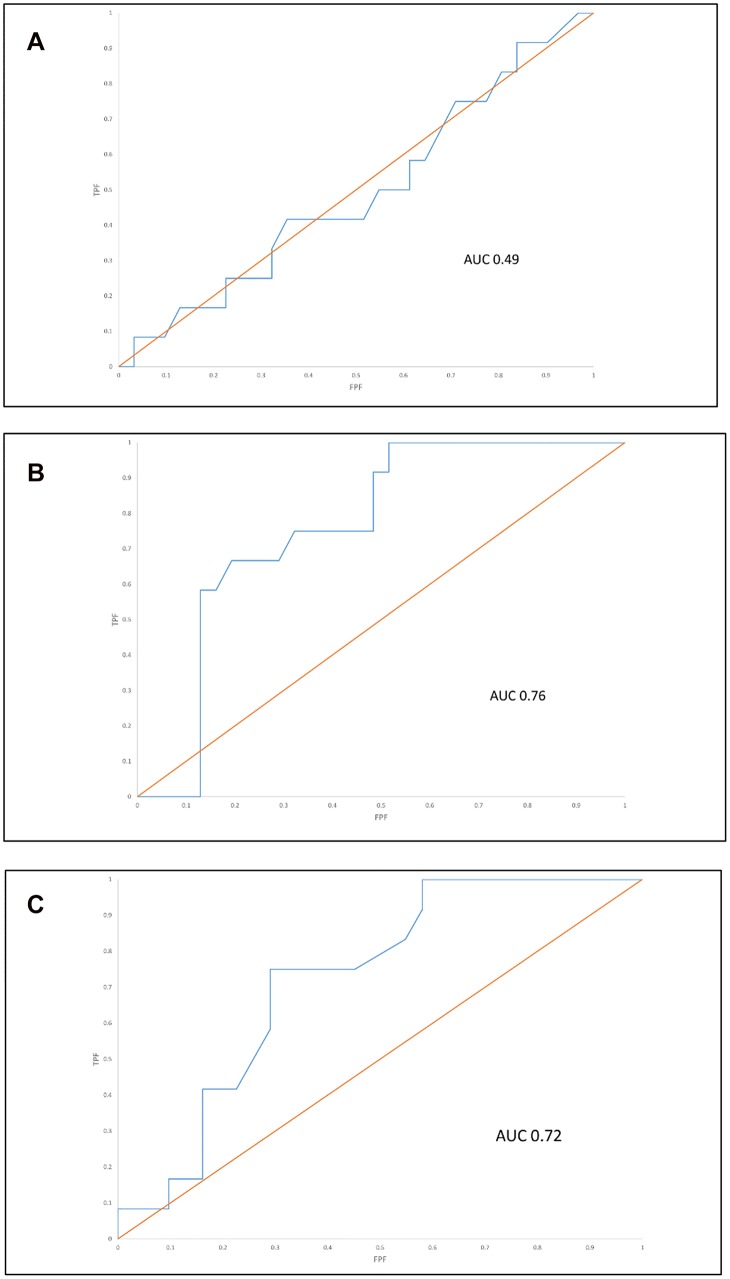
Receiver-operating curves for the outcome of operative delivery (emergency CS or operative vaginal delivery) for suspected fetal compromise among those who underwent labour (n = 12/43) using customised birthweight centile (Fig 4a), change in fetal weight centile between 28–36 weeks (Fig 4b) and change in fetal weight centile between 28 weeks-birth (Fig 4c).

Of the 48 participants, 5 (9.6%) underwent elective caesarean section and 43 (89.6%) underwent labor. Of these, 12/ 43 (28%) required operative delivery (ventouse, forceps or emergency caesarean section) for suspected intrapartum fetal compromise. The change in fetal weight centile, both between 28–36 weeks and 28 weeks-birth was compared to birthweight centile alone for predicting operative delivery for suspected intrapartum fetal compromise. Fig 4 provides ROC curves for the performance of birthweight centile (Fig 4a), changing weight centile between 28–36 weeks (Fig 4b), and changing weight centile between 28 weeks and birth (Fig 4c) for the outcome of operative delivery for suspected intrapartum fetal compromise. The area under the curve (AUC), both for change in centile between 28 and 36 weeks (AUC 0.76), and 28 weeks and birth (AUC 0.72), was significantly higher than for birthweight centile (AUC 0.49), p <0.01 and p = 0.02, respectively.

For *slowing growth between 28 and 36 weeks*, the optimal threshold for predicting intrapartum compromise was a fall in fetal weight centile of ≥ 10, occurring in 8/14 with slowing growth, compared to 4/29 with maintained growth (p = 0.009; sensitivity 57%, specificity 86%, PPV 67% and NPV 81%). For *slowing growth between 28 weeks and birth*, the optimal threshold for predicting intrapartum compromise was a fall in fetal weight centile of ≥ 20 (ie. Group 2 and 3 combined) occurring in 9/18 (50%) with *slowing growth*, compared to 3/25 (12%) cases with *maintained growth* (p = 0.01; sensitivity 50%, specificity 88%, PPV 75%, NPV 71%). S1 Table summarises the test characteristics by varying thresholds across both time epochs.

Cord pH is not routinely collected at all deliveries in our hospital, but was obtained as often as possible for study participants. This was available for 29/48 (69%) women in this study. The incidence of cord pH ≤ 7.2 was 4/17 (24%) among those with maintained growth from 28 weeks-birth, 2/5 (40%) among those with a moderate decline in growth, and 2/7 (33%) among those with a severe decline in growth, a trend that was not statistically significant (p = 0.61).

## Discussion

We have performed a prospective longitudinal study and have obtained both ultrasound and clinical evidence suggesting that AGA fetuses who demonstrate slowing of growth in late pregnancy may be experiencing placental insufficiency. We have observed a significant continuous relationship between a fall in fetal growth centile across the third trimester and falling cerebral vascular resistance. Despite the modest regression coefficients, the stepwise fall in MCA-PI among those with slowing growth compared to those that maintained growth velocity suggests that such growth decrements may not be benign, and result in subtle but significant changes in adaptive fetal physiology. These associations were seen for both the MCA-PI and cerebroplacental ratio. Although few measurements were under the 5^th^ centile, this finding is consistent with other reports in unselected populations which have found this dichotomous threshold performs poorly [14], while a composite score incorporating Doppler centiles performs better [7], as a predictor of adverse outcomes due to presumed placental insufficiency. In addition to more cerebral vasodilation, we observed that fetuses with slowing of growth in late pregnancy were more likely to become compromised when faced with the hypoxic challenge of labour, a hallmark of pregnancies with FGR due to placental insufficiency [8, 15, 16]. This study suggests that vigilance for late fetal growth slowing- right up until the time of delivery- may be important. While the small numbers mean these findings must be interpreted with caution, the predictive performance of fetal growth trajectory for intrapartum fetal compromise is encouraging and warrants further investigation.

That fetal growth slowing in those otherwise born AGA is associated with features of placental insufficiency is perhaps not surprising. In the face of placental insufficiency, fetal growth slows, but whether the birthweight reaches < 10^th^ centile is likely to depend upon the starting fetal weight centile, the trajectory of the relative slowing in growth and gestational age at delivery. Previous studies have reported that slowing of fetal growth is associated with increased risks of adverse perinatal outcome. These found a decline in growth trajectory was associated with emergency delivery for fetal distress, umbilical artery cord pH <7.1 and admission to neonatal intensive care [17–21]. However, these previous studies included cases of SGA, where the birthweight was <10^th^ centile at delivery. We deliberately examined a cohort that was exclusively AGA at birth. Our findings are consistent with several large population based cohorts reporting the lowest risk of perinatal death in infants with birthweight between the 75^th^ and 90^th^ centile. The stepwise increase in perinatal death with falling birthweight centile may be partly explained by placental insufficiency causing slowing of growth, even among those AGA at birth [22].

The implication from this study is that fetuses that slow in growth in utero, but are born AGA (and not classed as growth restricted) may represent a population at increased risk of adverse fetal outcomes, including stillbirth. It is possible such cases may benefit from increased fetal surveillance in late pregnancy and in labour, and it may even be prudent to offer induction at term. It is premature to introduce such fetal surveillance into the clinic, but we believe our data has identified an under-appreciated ‘at risk’ cohort that merits further prospective investigation in large and appropriately powered cohorts. Epidemiologically, it could be argued that identifying ‘at risk’ AGA fetuses in pregnancy is even more important than identifying those fetuses that are SGA: a recent study involving over 6,000 infants confirmed that- while the incidence of adverse perinatal outcomes is higher in SGA than AGA fetuses- the majority of adverse perinatal outcomes (including stillbirth, low cord pH, emergency caesarean delivery for fetal distress in labour and neonatal intensive care unit admission) occur among those AGA [14]. It is an exciting possibility that better identification of AGA feuses at risk would enable improved surveillance and timely delivery, resulting in a similar reduction in stillbirth risk to that observed with antenatal detection of SGA [2].

The correlations observed between the fall in fetal growth centile and the MCA-PI and cerebroplacental ratio measured at 36 weeks were not observed for the UA-PI. This is not unexpected; while umbilical artery Doppler has an established place in the management of high risk pregnancies [23], and early onset FGR [24], adverse events due to FGR in late pregnancy are rarely associated with reduced umbilical artery flow [5]. However, centralisation of the fetal circulation is associated with an increased risk of adverse perinatal outcome in late preterm and term FGR [25–30], and this study adds to the growing evidence surrounding the role of cerebral dopplers in late pregnancy among the apparently well grown [7, 14, 31].

Despite the limitation of small numbers, our study had a number of strengths. It was prospective in design and included longitudinal recording of fetal growth by one experienced operator in an otherwise low risk cohort. We reported fetal weight centile using a customised intrauterine fetal growth curve [9], rather than using population, or live birthweight, centiles. Population charts will inevitably report a higher weight centile for preterm fetuses still *in utero*, since these fetuses are being compared to delivered newborns, a cohort subject to a range of pathologies that impair growth. In assessing fetal growth trajectory, it is important to use an appropriate *in utero* comparator, since many fetuses will appear to slow in their growth if using population birthweight centiles as a reference standard. A limitation of this study is the relatively small sample size and that we were unable to obtain placental histology, or umbilical cord pH on all cases that had laboured.

## Conclusion

Slowing of fetal growth trajectory in AGA fetuses may be an important indicator of placental insufficiency, providing a potential explanation for a portion of stillbirths occurring in the AGA population. If confirmed, evaluation of growth trajectory may usefully add to the assessments of placental reserve and fetal well-being currently employed in an attempt to decrease the incidence of late pregnancy stillbirth.

## Supporting Information

S1 Dataset Minimal summary dataset for the cohort. (XLS)

S1 Table Predictive test characteristics of changing centile as a predictor of operative delivery (Em CS or OVD) for suspected intrapartum fetal compromise; n = 12/43. (DOCX)
