# Supplementary material for: Placental Insufficiency in Fetuses That Slow in Growth but Are Born Appropriate for Gestational Age: A Prospective Longitudinal Study
Source: PLoS One. 2016 Jan 5;11(1):e0142788. doi: 10.1371/journal.pone.0142788 (PMC4701438; doi:10.1371/journal.pone.0142788)
Supplement: S1 Table — (DOCX) [file pone.0142788.s002.docx]

**S1 Table:** Predictive test characteristics of changing centile as a predictor of operative delivery (Em CS or OVD) for suspected intrapartum fetal compromise; n= 12/43

| Test Threshold | Sensitivity | Specificity | Positive predictive value | Negative predictive value | P value |
| --- | --- | --- | --- | --- | --- |
| **Change in Fetal Weight Centile Between 28 and 36 weeks** | | | | | |
| Change in fetal weight centile ≥ 10 (n=14; 33%) | 57% | 86% | 67% | 81% | 0.009** |
| Change in fetal weight centile ≥ 20 (n=5; 12%) | 20% | 71% | 8% | 87% | 1.0 |
| Change in fetal weight centile ≥ 30 (n=2; 5%) | 0 | 94% | 0 | 71% | 1.0 |
| **Change in Fetal Weight Centile Between 28 weeks and Birth** | | | | | |
| Change in fetal weight centile ≥ 10 (n=23; 53%) | 39% | 85% | 75% | 55% | 0.09 |
| Change in fetal weight centile ≥ 20 (n=18; 42%) | 50% | 88% | 75% | 71% | 0.01* |
| Change in fetal weight centile ≥ 30 (n=11; 26%) | 45% | 78% | 42% | 81% | 0.24 |
